# A low-crystalline ruthenium nano-layer supported on praseodymium oxide as an active catalyst for ammonia synthesis†
†Electronic supplementary information (ESI) available: Detailed procedures for each method, catalytic performance, STEM-EDX images, detailed characterization. See DOI: 10.1039/c6sc02382g


**DOI:** 10.1039/c6sc02382g

**Published:** 2016-09-19

**Authors:** Katsutoshi Sato, Kazuya Imamura, Yukiko Kawano, Shin-ichiro Miyahara, Tomokazu Yamamoto, Syo Matsumura, Katsutoshi Nagaoka

**Affiliations:** a Elements Strategy Initiative for Catalysts and Batteries , Kyoto University , 1-30 Goryo-Ohara, Nishikyo-ku , Kyoto 615-8245 , Japan; b Department of Applied Chemistry , Faculty of Engineering , Oita University , 700 Dannoharu , Oita 870-1192 , Japan . Email: nagaoka@oita-u.ac.jp; c Department of Applied Quantum Physics and Nuclear Engineering , Kyushu University , 744 Motooka, Nishi-ku , Fukuoka 819-0395 , Japan

## Abstract

Ammonia is a crucial chemical feedstock for fertilizer production and is a potential energy carrier. However, the current method of synthesizing ammonia, the Haber–Bosch process, consumes a great deal of energy. To reduce energy consumption, a process and a substance that can catalyze ammonia synthesis under mild conditions (low temperature and low pressure) are strongly needed. Here we show that Ru/Pr_2_O_3_ without any dopant catalyzes ammonia synthesis under mild conditions at 1.8 times the rates reported with other highly active catalysts. Scanning transmission electron micrograph observations and energy dispersive X-ray analyses revealed the formation of low-crystalline nano-layers of ruthenium on the surface of Pr_2_O_3_. Furthermore, CO_2_ temperature-programmed desorption revealed that the catalyst was strongly basic. These unique structural and electronic characteristics are considered to synergistically accelerate the rate-determining step of NH_3_ synthesis, cleavage of the N≡N bond. We expect that the use of this catalyst will be a starting point for achieving efficient ammonia synthesis.

## Abstract 2

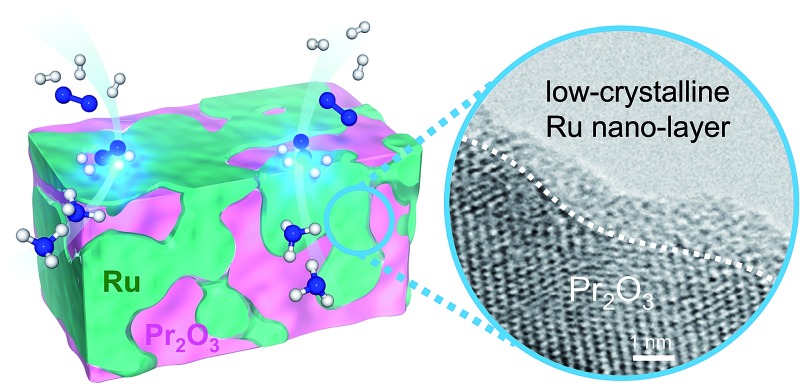
Low-crystalline Ru nano-layers and the strong basicity of Ru/Pr_2_O_3_ synergistically accelerated the rate-determining step of ammonia synthesis.

## Introduction

Ammonia is one of the most important feedstocks in the modern chemical industry. Globally, >80% of ammonia produced is used to produce fertilizer, which is essential for growing crops.^1^ In addition, ammonia has recently attracted attention as a carrier of energy and hydrogen.^2–5^ Ammonia is produced by combining atmospheric N_2_ with hydrogen produced by renewable energy. The ammonia is liquefied and transported to where it is used to generate power in engines or electricity in fuel cells. Ammonia is being considered as a carrier of energy and hydrogen because, (1) it has a high energy density (12.8 GJ m^–3^) and (2) a high hydrogen content (17.6 wt%), and (3) carbon dioxide is not released when hydrogen is produced by ammonia decomposition.^2^ If ammonia can be produced efficiently from renewable energy, it can contribute to the solution of global problems related to energy and food production.

Currently, most ammonia is synthesized *via* the Haber–Bosch process.^6–8^ This process is a major consumer of energy, accounting for about 1% of global energy consumption. In this process, about 60% of consumed energy is recovered and saved in ammonia as enthalpy. However, the remaining energy is lost, mainly during the production of hydrogen from natural gas, ammonia synthesis, and gas separation. Because ammonia synthesis is carried out at very high temperatures (>450 °C) and high pressures (>20 MPa), a major goal is the reduction of the high amount of energy used in this process.^9^ Curbing global energy consumption requires, *inter alia*, a catalyst that is able to produce ammonia at much lower temperatures and pressures than required for the iron-based catalysts used in the Haber–Bosch process.^10–12^

Ruthenium is a possible catalyst for ammonia synthesis because of its higher activity at low pressure and temperature compared to that of iron-based catalysts. The rate-determining step in NH_3_ synthesis is cleavage of the N≡N bond of N_2_, because the bond energy is very high (945 kJ mol^–1^).^13,14^ It has been reported that modification of the morphology of the Ru surface (“structural modification”) and of the Ru electronic states (“electronic modification”) are effective ways to accelerate the rate-determining step and thus enhance the ammonia-synthesis activity of the Ru catalyst.^15,16^ In the case of structural modification, the unusual unsaturated B_5_-type site of Ru has been proven to be highly active.^17–19^ The B_5_-type site consists of five Ru atoms: two at step edges and three on the lower terrace. The five Ru atoms are all associated with the transition state of adsorbed N_2_, which results in weakening of the N≡N bond.^17^ Adjusting the Ru particle size (*e.g.*, to 5 nm when Ru particles are spherical) and changing the shape of Ru particles create an abundance of B_5_-type sites.^18,20,21^ In the case of electronic modification, the use of basic supports and the addition of a strong basic promoter to Ru catalysts have enhanced ammonia synthesis activity dramatically.^15,16^ The mechanism involves the transfer of electrons to the Ru metal from the basic components. Transfer of electrons from Ru to the antibonding π-orbitals of N_2_ then results in weakening of the N≡N bond and promotion of N≡N cleavage.^22^ Weakening of the N≡N bond by doping with strong basic oxides has been confirmed by observation of the N≡N stretching frequency with infrared spectroscopy (IR); the most effective promoter has been reported to be Cs_2_O.^23,24^ In fact, most of the highly active Ru catalysts contain Cs_2_O as a promoter.^10,15,25,26^ However, CsOH, which may be produced in the presence of an H_2_O impurity in the reactant, has a low melting point (272 °C) and may move on the surface of the catalyst particles or vaporize under the reaction conditions, the eventual result being degradation of the catalyst.^27^ On the other hand, BaO is also reported as an effective promoter and Ba–Ru/activated carbon (Ba–Ru/AC) has been used in commercial industrial processes.^28^ Recently, Horiuchi *et al.* reported that Ru/BaTiO_3_ and Ba–Ru/MgO show comparable high activity to Cs–Ru/MgO.^26^ Notably, Ru-loaded electride [Ca_24_Al_28_O_64_]^4+^(e^–^)_4_ (Ru/C12A7:e^–^), which is a new class of Ru catalyst supported on a non-oxide, shows high NH_3_-synthesis activity without any dopant.^10,29,30^ This high activity has been attributed to the high electron-donating power of the electride.

We show here that a praseodymium oxide-supported Ru catalyst (Ru/Pr_2_O_3_) without any dopant exhibits unparalleled NH_3_ synthesis ability compared with highly active catalysts reported previously. The loading of Ru on the support was characterized by an unusual morphology of low-crystalline nano-layers, and the basicity of the catalyst was very high. We show that the combination of these features facilitated the activation of N_2_.

## Results and discussion

### NH_3_-synthesis activities of supported Ru-catalysts

**Fig. 1:**
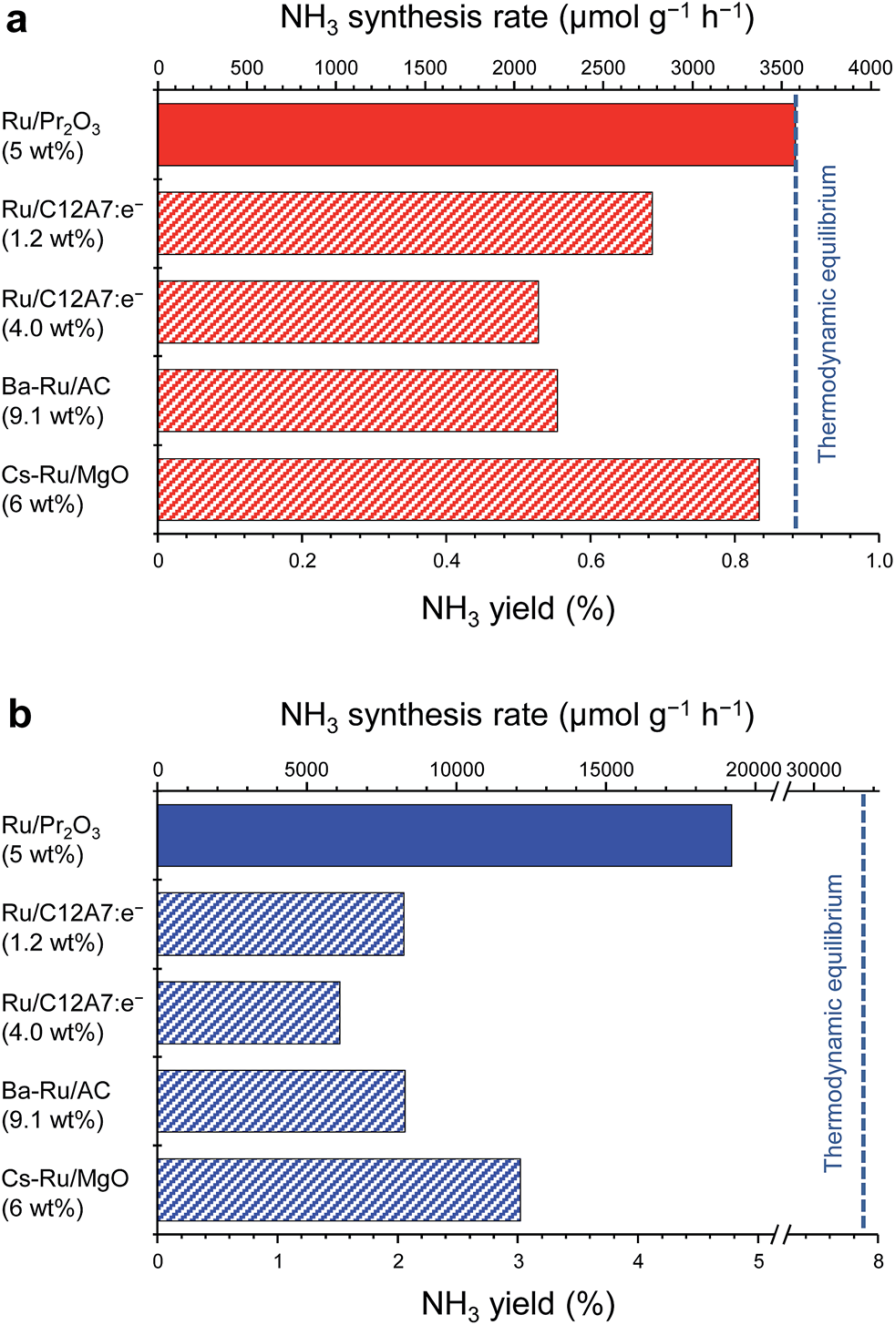
Catalytic performance of supported Ru catalysts for NH_3_ synthesis at (a) 0.1 MPa and (b) 1 MPa. Reaction conditions: catalyst, 0.2 g; reactant gas, H_2_/N_2_ = 3 with a flow rate of 60 mL min^–1^; reaction temperature, 400 °C. With the exception of Ru/Pr_2_O_3_, NH_3_ synthesis rates are reproduced from ref. 10.

Fig. 1 compares the NH_3_-synthesis activity of the Ru/Pr_2_O_3_ catalyst with that of other supported Ru catalysts under the same reaction conditions. Ba–Ru/activated carbon (Ba–Ru/AC) has been used in industrial processes;^28^ Cs–Ru/MgO is one of the most active Ru catalysts in NH_3_ synthesis;^25,31^ and Ru/C12A7:e^–^ has attracted attention as a new active NH_3_-synthesis catalyst.^10–12^ At 400 °C and 0.1 MPa (Fig. 1a), Ru/Pr_2_O_3_ and Cs–Ru/MgO gave NH_3_ yields near the thermodynamic equilibrium (0.88%). Both the yields and NH_3_ production rates were higher than those achieved with the Ru/C12A7:e^–^ and Ba–Ru/AC catalysts. In the industrial process, it is important to obtain high one-pass NH_3_ yields to avoid the high energy usage required for gas separation. Furthermore, from the standpoint of thermodynamic regulation, NH_3_ synthesis is favored if the reaction is carried out under high pressure.^9^ We therefore measured the NH_3_-synthesis activity at 1.0 MPa (Fig. 1b), where the NH_3_ yield at the thermodynamic equilibrium increases to 7.9%. Note that 1.0 MPa is still much lower than the reaction pressure used for the Haber–Bosch process. With the increase in reaction pressure, the differences in the activities of the catalysts were more pronounced: the NH_3_ yield reached 4.8% and the rate of formation obtained over Ru/Pr_2_O_3_ reached 19 000 μmol g^–1^ h^–1^, >1.8 times the values associated with other catalysts.

**Fig. 2:**
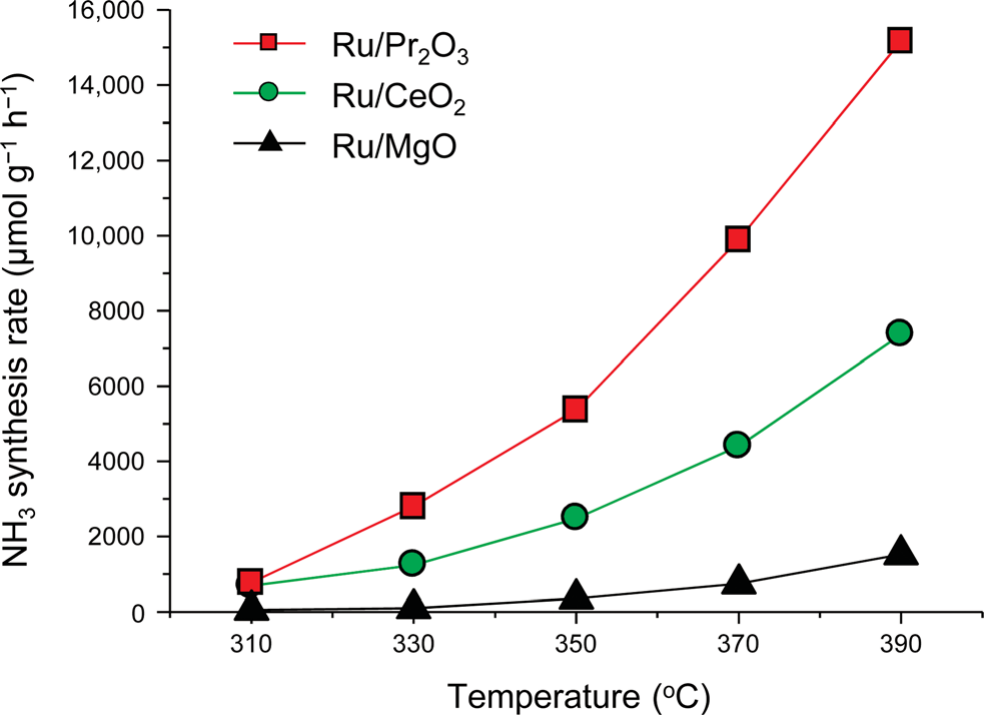
Rate of NH_3_ synthesis over supported Ru catalysts. Reaction conditions: catalyst, 0.2 g; reactant gas, H_2_/N_2_ = 3 with a flow rate of 60 mL min^–1^; pressure, 0.9 MPa.

To understand why the rates of NH_3_ synthesis are so high when catalyzed by Ru/Pr_2_O_3_, we compared the characteristics of Ru/Pr_2_O_3_ with those of Ru/MgO and Ru/CeO_2_. All of the catalysts were loaded with 5 wt% Ru. Among the dopant-free simple oxide-supported Ru catalysts, Ru/MgO and Ru/CeO_2_ have shown relatively high NH_3_-synthesis activity,^32^ and CeO_2_ is a rare-earth oxide like Pr_2_O_3_. Fig. S2† shows *in-situ* X-ray diffraction patterns of the catalysts after activation in pure H_2_ at 400 °C. In the cases of Ru/MgO and Ru/CeO_2_, only diffraction patterns assigned to cubic-type MgO and CeO_2_ were obtained. In the case of Ru/Pr_2_O_3_, the diffraction peaks were attributed to rare earth C-type Pr_2_O_3_.^33^ On the other hand, the fact that no diffraction peaks of the Ru species were apparent in the patterns of the catalyst samples suggests that the crystallite size of the loaded Ru was too small to be detected. NH_3_-synthesis activities of the Ru catalysts were then measured at 0.9 MPa after reduction at 400 °C. Ru/Pr_2_O_3_ catalyzed NH_3_ synthesis at a much higher rate than that of Ru/MgO and Ru/CeO_2_ at all temperatures from 310 to 390 °C (Fig. 2). At 390 °C in particular, the NH_3_ synthesis rate of Ru/Pr_2_O_3_ was 15 200 μmol g^–1^ h^–1^, much higher than that of Ru/CeO_2_ (7400 μmol g^–1^ h^–1^) and Ru/MgO (1500 μmol g^–1^ h^–1^). Furthermore, the long-term stability of the Ru/Pr_2_O_3_ catalyst at 390 °C under 0.9 MPa was evidenced by the fact that the rate of NH_3_ synthesis was stable for 50 h (Fig. S3†).

Specific surface areas of Ru/Pr_2_O_3_, Ru/CeO_2_, and Ru/MgO were 20.4, 33.5, and 46.4 m^2^ g^–1^, respectively (Table 1). There was no clear correlation between specific surface area and catalytic activity. Interestingly, the H/Ru ratio, a measure of Ru dispersion, was very low for Ru/Pr_2_O_3_ compared with that of the other catalysts. As a result, the turnover frequency of Ru/Pr_2_O_3_ was >3.5 times that of Ru/CeO_2_ and Ru/MgO. These results suggest that the high turnover frequency of Ru/Pr_2_O_3_ makes the excellent rate of synthesis of NH_3_ (activity per weight of catalyst) possible.

**Table 1:** Physicochemical properties of supported Ru catalysts

Catalyst	Specific surface area (m^2^ g^–1^)	H/Ru^*a*^	Turnover frequency^*b*^ (s^–1^)	Density of base sites^*c*^ (μmol m^–2^)
Ru/Pr_2_O_3_	20.4	0.17	0.050	4.4
Ru/CeO_2_	33.5	0.29	0.014	2.3
Ru/MgO	46.4	0.3	0.003	2.2

^*a*^Estimated by using H_2_ chemisorption capacity.

^*b*^Calculated by using H/Ru value and NH_3_ yield at 390 °C under 0.9 MPa.

^*c*^Estimated by using CO_2_-TPD.

### Structural properties of Ru/Pr_2_O_3_

**Fig. 3:**
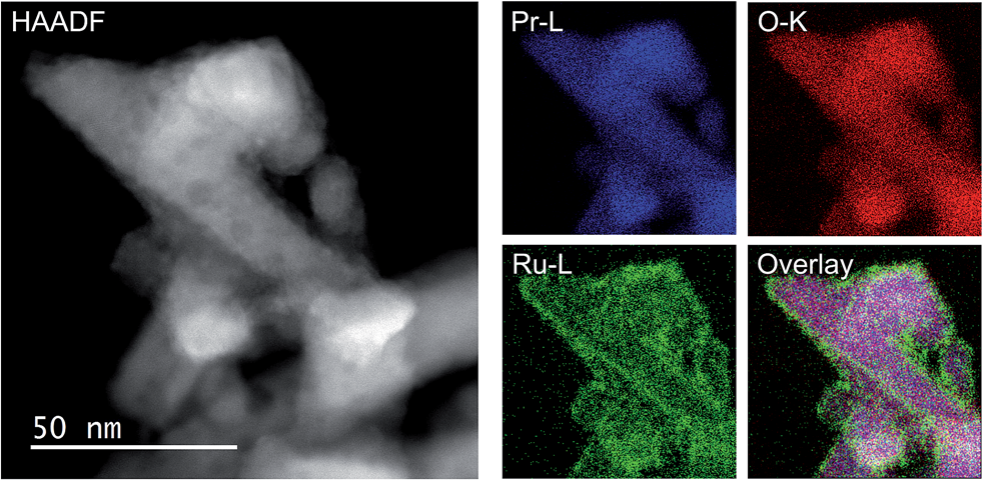
HAADF-STEM image, Pr-L, O-K, and Ru-L STEM-EDX maps, and reconstructed overlay image of Pr, O, and Ru for Ru/Pr_2_O_3_ after H_2_ reduction.

**Fig. 4:**
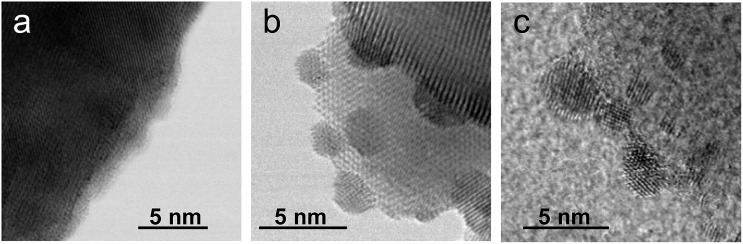
HR-STEM images of (a) Ru/Pr_2_O_3_, (b) Ru/CeO_2_, and (c) Ru/MgO, after H_2_ reduction.

As the NH_3_-synthesis ability of a supported Ru catalyst is related to the morphology of the loaded Ru and the basicity of the support material, we used scanning transmission electron micrograph (STEM) observations and energy dispersive X-ray (EDX) analysis to investigate the morphology. Fig. 3 and S4† show high-angle annular dark-field (HAADF) images and EDX maps of Ru/Pr_2_O_3_ following treatment of the catalyst with H_2_ at 400 °C. Fig. S5 and S6† show analogous images and maps of Ru/CeO_2_ and Ru/MgO, respectively. A number of particles identified as Ru species by EDX were supported on MgO and CeO_2_, but were seldom observed over Pr_2_O_3_. However, the EDX map showed that Ru was dispersed over the entire Pr_2_O_3_ surface. In the reconstructed overlapping EDX images, the greenish edges of the catalyst particles indicated that the surfaces of the catalyst particles were covered by the Ru species. These results suggest that the state of Ru is completely different when it is loaded over Pr_2_O_3_ *versus* MgO and CeO_2_. To further investigate the surface morphology, we made high-resolution STEM (HR-STEM) observations (Fig. 4, and see Fig. S7–S9†). On Ru/MgO and Ru/CeO_2_, the lattice fringes of the Ru species and the supports were clearly apparent. The *d* space of the Ru species was 0.21 nm, which is consistent with that of the (101) plane of metallic Ru. Mean diameters of the Ru particles were 1.8 ± 0.7 nm on Ru/MgO and 2.5 ± 0.8 nm on Ru/CeO_2_. In addition, the surface of the supports of these catalysts was smooth, and changes in the lattice fringe were clearly observed on the boundaries between Ru particles and supports (Fig. 4b and c, S8 and S9†). In contrast, on Ru/Pr_2_O_3_, the surface of Pr_2_O_3_ was covered by layers of Ru rather than by particles. The fact that the lattice fringes of most parts of the Ru layers were not apparent indicated that the crystallinity of the Ru layers was low. The thickness of the Ru layers was 0.5–3 nm, and Ru particles were sometimes included in the layers. Thus, we considered that the surface of Pr_2_O_3_ was covered mainly with low-crystalline Ru nano-layers.

To explain why the Ru on the Pr_2_O_3_ support possessed such a unique morphology, we analysed the X-ray diffraction patterns of the catalyst precursors of Ru/Pr_2_O_3_. As shown in Fig. S10,† the bare support [before impregnation with Ru_3_(CO)_12_] showed the structure of fluorite-type Pr_6_O_11_. However, after impregnation with Ru_3_(CO)_12_ in tetrahydrofuran (THF) and drying, the peaks assigned to Pr_6_O_11_ became smaller, and peaks attributed to Pr(OH)_3_ and PrOOH appeared. Furthermore, after heat treatment under a stream of Ar at 350 °C, only peaks corresponding to PrOOH were observed. At this point, the HAADF STEM and overlay of the EDX maps of Ru/Pr_2_O_3_ demonstrated that the surfaces of the catalyst particles were covered by Ru species (Fig. S11†). These results indicate that Ru_3_(CO)_12_ reacted with the O^2–^ in Pr_6_O_11_ and Pr^4+^ was reduced to Pr^3+^, with the formation of CO_2_. The support then reacted with the H_2_O impurity in the THF, and after heat treatment in the Ar stream, Ru and PrOOH were formed. In brief, the results reveal that the high reactivity between Ru_3_(CO)_12_ and Pr_6_O_11_ prevented aggregation of Ru_3_(CO)_12_ with Ru_3_(CO)_12_ and contributed to the formation of the unique structure of the loaded Ru. The rough surface of the Pr_2_O_3_ and the fuzziness of the boundary between Ru and Pr_2_O_3_ in the HR-STEM image in Fig. 4a and S7† was probably due to the reaction between Ru_3_(CO)_12_ and Pr_6_O_11_. Furthermore, during H_2_ treatment, PrOOH was converted to Pr_2_O_3_ (Fig. S2†). During this process, part of the Ru included in the Ru layers was crystallized to form Ru particles, and thus Ru particles were sometimes observed in the Ru layers in the HR-STEM images (Fig. S7†). As shown in the HR-STEM images, the Ru species over Pr_2_O_3_ were arranged in a low-crystalline, nano-layered structure. In such a structure, unsaturated Ru atoms were not precisely arranged and formed step-and-terrace sites similar to a B_5_-type site. The unique surface morphology of Ru in Ru/Pr_2_O_3_ would promote N_2_ adsorption and subsequent cleavage of the N≡N bond.

In addition, we carried out STEM-EDX observations of Ru/Pr_2_O_3_ after the long-term stability test shown in Fig. S3.† As shown in Fig. S12 and S13,† Pr_2_O_3_ was still covered with low-crystalline Ru nano-layers, as it was before reaction, and distinct changes of the structure were not observable. These results demonstrate the high durability of the unique surface structure of Ru/Pr_2_O_3_ under the conditions used for NH_3_ synthesis.

### Basic properties of Ru/Pr_2_O_3_

**Fig. 5:**
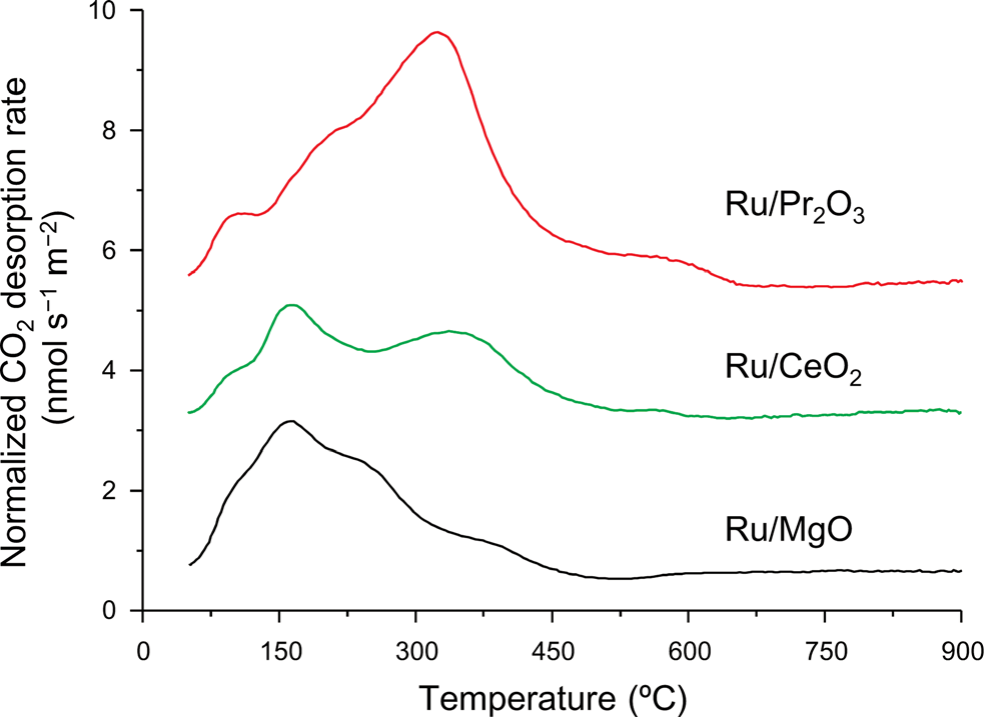
CO_2_-TPD profiles of supported Ru catalysts. Following H_2_ reduction at 400 °C, CO_2_ adsorption was carried out at 50 °C. These curves show the difference between the curves shown in Fig. S14† to remove the contribution of CO_2_ that remained on the surface of the catalysts even after H_2_ pre-treatment.

We used CO_2_ temperature-programmed desorption (CO_2_-TPD) measurements of the catalysts (Fig. 5) to evaluate another crucial determinant of NH_3_-synthesis ability, the basicity of the support. To remove the contribution of the CO_2_ that remained on the surface even after H_2_ reduction, we subtracted the CO_2_-TPD profile without CO_2_ adsorption from that after CO_2_ adsorption (see Fig. S14† for original figures). CO_2_ desorption was observed at 50–680 °C on Ru/Pr_2_O_3_, 50–600 °C on Ru/CeO_2_, and 50–500 °C on Ru/MgO. CO_2_ desorption observed in the high temperature region (≥300 °C) was greatest on Ru/Pr_2_O_3_, intermediate on Ru/CeO_2_, and least on Ru/MgO. These results indicate that the basic sites on Ru/Pr_2_O_3_ are the strongest, and those on Ru/MgO are the weakest. We used the total amount of CO_2_ desorbed as a metric of basic density over the catalysts. Ru/Pr_2_O_3_ had the highest basic density, 4.4 μmol m^–2^, almost twice that of Ru/CeO_2_, 2.3 μmol m^–2^, and Ru/MgO, 2.2 μmol m^–2^. These results reveal that the surface basicity of Ru/Pr_2_O_3_ was much stronger than that of Ru/MgO and Ru/CeO_2_. This strong surface basicity results in the most effective electron donation to Ru and promotes N_2_ adsorption and subsequent cleavage of the N≡N bond. Furthermore, we can say that Pr_2_O_3_ is covered by islands of Ru nano-layers, which allow large amounts of CO_2_ to adsorb on the surface of uncovered Pr_2_O_3_. Note also that the CO_2_ desorption temperature and the total density of the basic sites were higher on Ru/CeO_2_ than on Ru/MgO. This difference accounts for the higher NH_3_-synthesis activity of Ru/CeO_2_ than that of Ru/MgO.

### Activation of N_2_ over Ru/Pr_2_O_3_

**Fig. 6:**
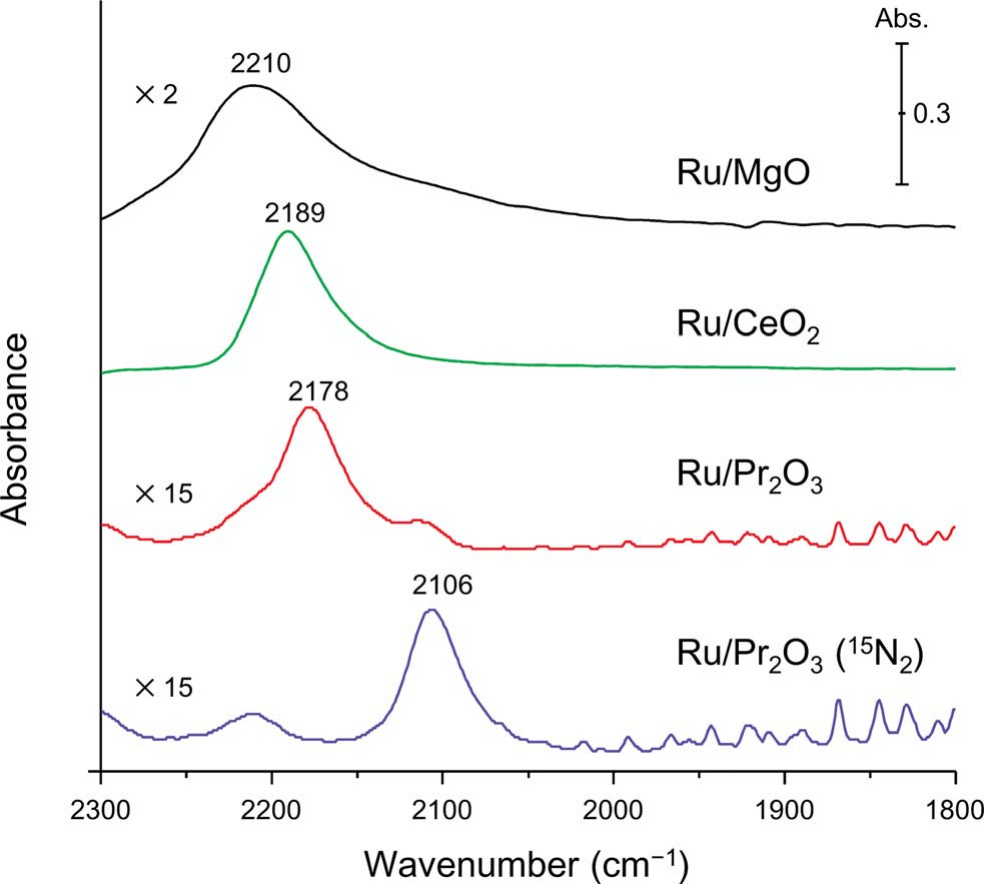
Difference infrared spectra of N_2_ molecules (before and after N_2_ adsorption) on supported Ru catalysts. Spectra were collected under 6 kPa of N_2_ (^15^N_2_ for Ru/Pr_2_O_3_) at 25 °C.

Finally, to understand the activation of N_2_ molecules over the Ru/Pr_2_O_3_ catalyst, we examined the states of the adsorbed N_2_ with FT-IR techniques. The IR spectra after the addition of N_2_ to Ru/MgO, Ru/CeO_2_, and Ru/Pr_2_O_3_ at room temperature are shown in Fig. 6. The IR spectrum of each catalyst shows a broad peak around 2350 to 2100 cm^–1^; such peaks are assignable to the stretching vibration mode of the N_2_ adsorbed with an end-on orientation on the Ru surface.^21,23,24^ Note that the peak absorbance of N_2_ adsorbed on Ru/Pr_2_O_3_ occurred at a lower frequency (2178 cm^–1^) than the corresponding peak absorbances on Ru/MgO (2210 cm^–1^) and Ru/CeO_2_ (2189 cm^–1^). In the spectrum of ^15^N_2_ adsorbed on Ru/Pr_2_O_3_, the peak absorbance was shifted to a lower frequency (2106 cm^–1^) compared to that on Ru/Pr_2_O_3_ (2178 cm^–1^), which is in good agreement with the frequency estimated from the isotope effect (2178 cm^–1^ × (14/15)^1/2^ = 2104 cm^–1^).^23,24^ These results suggest that these peaks are associated with the N_2_ on the Ru surfaces. The lower frequencies of the peak absorbances of N_2_ adsorbed on Ru/Pr_2_O_3_ compared to those of Ru/MgO and Ru/CeO_2_ indicate that the N≡N bond of N_2_ was further weakened over the low-crystalline Ru nano-layers on Pr_2_O_3_ relative to Ru nanoparticles on the other supports. We surmise that the morphology of the Ru surface and the basicity of the catalyst contributed synergistically to the weakening of the N≡N bond and enhanced the catalytic activity for NH_3_ synthesis.

## Conclusions

In summary, we demonstrated that Ru/Pr_2_O_3_ without any dopant catalyzed a high rate of NH_3_ synthesis under mild reaction conditions (0.1–1.0 MPa). Characteristics of Ru/Pr_2_O_3_ include low-crystalline Ru nano-layers formed by the reaction between Ru_3_(CO)_12_ and Pr_6_O_11_ and strong basicity of Pr_2_O_3_. These characteristics are considered to synergistically accelerate the rate-determining step of ammonia synthesis: cleavage of the N≡N bond of N_2_. In addition, substitution of some of the praseodymium with another element without degrading its activity for NH_3_ synthesis is currently in progress, because Pr is an expensive element. The outcome of the research will appear in a coming contribution. We believe that our catalyst will facilitate the development of an effective method for synthesizing ammonia from renewable energy under environmentally benign conditions. Such a method can be expected to contribute to the solution of food and energy crises globally.
