# Supplementary material for: A low-crystalline ruthenium nano-layer supported on praseodymium oxide as an active catalyst for ammonia synthesis
Source: Chem Sci. 2016 Sep 19;8(1):674–9. doi: 10.1039/c6sc02382g (PMC5297937; doi:10.1039/c6sc02382g)
Supplement: Supplementary file 1 [file SC-008-C6SC02382G-s001.pdf]

Electronic Supplementary Material (ESI) for Chemical Science.
This journal is © The Royal Society of Chemistry 2016

Electronic Supplementary Information

A low-crystalline ruthenium nano-layer supported on praseodymium oxide as an active catalyst for ammonia synthesis

Katsutoshi Sato,*[a,b] Kazuya Imamura,[b] Yukiko Kawano,[b] Shin-ichiro Miyahara,[a] Tomokazu Yamamoto,[c] Syo Matsumura,[c] and Katsutoshi Nagaoka*[b]

[a] Elements Strategy Initiative for Catalysts and Batteries, Kyoto University. 1-30 Goryo-Ohara, Nishikyo-ku, Kyoto 615-8245 (Japan)

[b] Department of Applied Chemistry, Faculty of Engineering, Oita University. 700 Dannoharu, Oita 870-1192 (Japan)

[c] Department of Applied Quantum Physics and Nuclear Engineering, Kyushu University. 744 Motooka, Nishi-ku, Fukuoka 819-0395 (Japan)

1. Details of experimental methods

1.1 Catalyst preparation

The Pr_6O_{11} support was prepared by precipitation at room temperature from a suspension formed by gradual addition of a solution of $Pr(NO_3)\cdot 6H_2O$ (Kanto Chemical, Japan) to a 25 wt% NH_3 solution (Wako Pure Chemical, Japan). The precipitate was kept in suspension overnight with stirring, washed with distilled water, dried at 70 °C for more than 12 h, and calcined at 700 °C in static air for 5 h. CeO_2 prepared by the same precipitation methods using $Ce(NO_3)_3\cdot 6H_2O$ (Wako Pure Chemical, Japan) and MgO (the reference catalyst of the Catalysis Society of Japan, JRC-MgO-500) were also calcined at 700 °C. The supports were then impregnated with $Ru_3(CO)_{12}$ (Tanaka Kikinzoku Kogyo, Japan) in a tetrahydrofuran (THF, Wako Pure Chemical, Japan) solution. The Ru loading was fixed at 5 wt% for each catalyst. The $Ru_3(CO)_{12}$-THF-support suspension was stirred for 12 h and dried in a rotary evaporator. The obtained powder was kept at 70 °C for 4 h under air. It was heated to 350 °C under a Ar stream and kept at 350 °C for 5 h to remove the CO ligand from the $Ru_3(CO)_{12}$.

1.2 Activity tests

The NH_3 synthesis rate was measured using a conventional flow system under either atmospheric pressure or high pressure. Powders of catalysts were pressed into pellets at 2.0 MPa for 5 min, crushed, and sieved to grains with diameters of 250–500 µm. Quartz wool was packed into a tubular Inconel reactor (i.d. = 7 mm), and 200 mg of catalyst was loaded. Research-grade gas was supplied from high-pressure gas cylinders. The catalysts were reduced in pure H_2 flow at 400 or 500 °C for 1 h at 0.1 MPa and then cooled to 310 °C in an Ar stream, and the pressure was then adjusted to 0.1, 0.9, or 1.0 MPa at 310 °C. An H_2/N_2 gas mixture with an H_2/N_2 molar ratio of 3 (gas hourly space velocity = 18,000 mL h^{-1} g^{-1}) was then fed to the catalyst. The temperature of the catalyst was kept constant for 0.5 h to facilitate measurement of NH_3 synthesis rates. The catalyst was then heated in 20 °C increments to 390 °C. The NH_3 synthesis rate was determined from the rate of decrease of electron conductivity (CM-30R, DKK-TOA, Japan) of the dilute sulfuric acid solution that trapped the NH_3 produced under the experimental conditions. NH_3 yield was calculated as described below:

$$\mathrm{NH_3\ yield\ (\%)} = \frac{F_{NH_3}}{inF_{N_2} \times 2} \times 100$$

where F_{NH_3} is molar flow rate of synthesized ammonia in effluent gas and inF_{N_2} is molar flow rate of N_2 which is supplied to catalyst. HSC Chemistry 6 software (ver. 6.12, Outotec Research, Finland) was used to calculate thermodynamic equilibrium.

1.3 Characterization of the catalysts

X-ray diffraction (XRD) analysis was performed using a SmartLab x-ray diffractometer (Rigaku, Japan) equipped with a Cu-Kα radiation source. For *in situ* XRD measurements, the sample was placed in a reactor chamber (XRK 900, Anton Parr) and treated at 400 °C for 1 h under a stream of

H_2. After treatment, the gas was switched from H_2 to N_2, the sample was cooled to room temperature, and diffraction patterns were obtained. PDXL2 software (Rigaku) with ICDD, COD,[S1] and AtomWork[S2] databases was used to analyze the XRD patterns.

High-angle, annular, dark-field, scanning transmission electron microscopy (HAADF-STEM) and high-resolution STEM (HR-STEM) images were obtained on a JEM-ARM200F electron microscope (JEOL, Japan) operated at 200 kV. The samples were dispersed in ethanol, dropped onto a carbon-coated copper grid, and dried under vacuum at ambient temperature for 24 h.
The specific surface areas of the catalysts after N_2 treatment at 300 °C were determined by the Brunauer–Emmett–Teller method using a BEL-mini instrument (BEL Japan Inc., Japan).
The H_2 chemisorption capacity was measured to estimate the Ru dispersion of the catalysts. H_2 was fed to each sample at 30 mL min^{-1}, and the temperature was increased to 400 °C. The sample was maintained at 400 °C for 1 h, purged in a stream of Ar at 500 °C for 30 min, cooled to –78 °C, and flushed with Ar for 60 min. After this pretreatment, H_2 chemisorption was carried out at –78 °C in an Ar stream (30 mL min^{-1}) using a pulsed-chemisorption technique.

Temperature-programmed desorption (TPD) measurements of CO_2 were performed in a TPD-1-AT apparatus (BEL Japan, Japan). Catalyst (100 mg) was loaded into a quartz reactor, reduced in a stream of H_2 at 400 °C for 1 h, purged in a stream of He for 30 min, and cooled to 50 °C. After 1% CO_2 in He gas (30 mL min^{-1}) was fed to the catalyst for 30 min at 50 °C, the oven temperature was increased at 10 °C min^{-1} to 900 °C. The CO_2 desorption profile was monitored by quadrupole mass spectrometer at *m/e* = 44. CO_2-TPD of catalysts not exposed to CO_2 at 50 °C was also measured.

The infrared spectra of adsorbed N_2 were collected by spectrometer (FT/IR-6600, Jasco, Japan) equipped with a mercury–cadmium–tellurium detector at a resolution of 4 cm^{-1}. Samples were pressed into self-supporting disks (10 mm diameter, about 20 mg). A disk was placed in a silica-glass cell equipped with CaF_2 windows and connected to a closed gas-circulation system. The disk was pretreated with circulated H_2 (26 kPa) passed through a liquid-nitrogen trap. The sample was heated from room temperature to 500 °C over 1 h and kept at that temperature for 3 h. Following reduction, the sample was evacuated at the same temperature for 2 h to remove the hydrogen. After this pretreatment, the disk was cooled to room temperature under vacuum. Pure N_2 (>99.9995%) was supplied to the system through a liquid-nitrogen trap. Isotopic nitrogen ($^{15}N_2$, 98%) was used without purification. The infrared spectrum of the sample at room temperature before N_2 adsorption was used as the background, and difference spectra were obtained by subtracting the backgrounds from the spectra of N_2-adsorbed samples.

2. Supporting Results

2.1. Ammonia synthesis performance

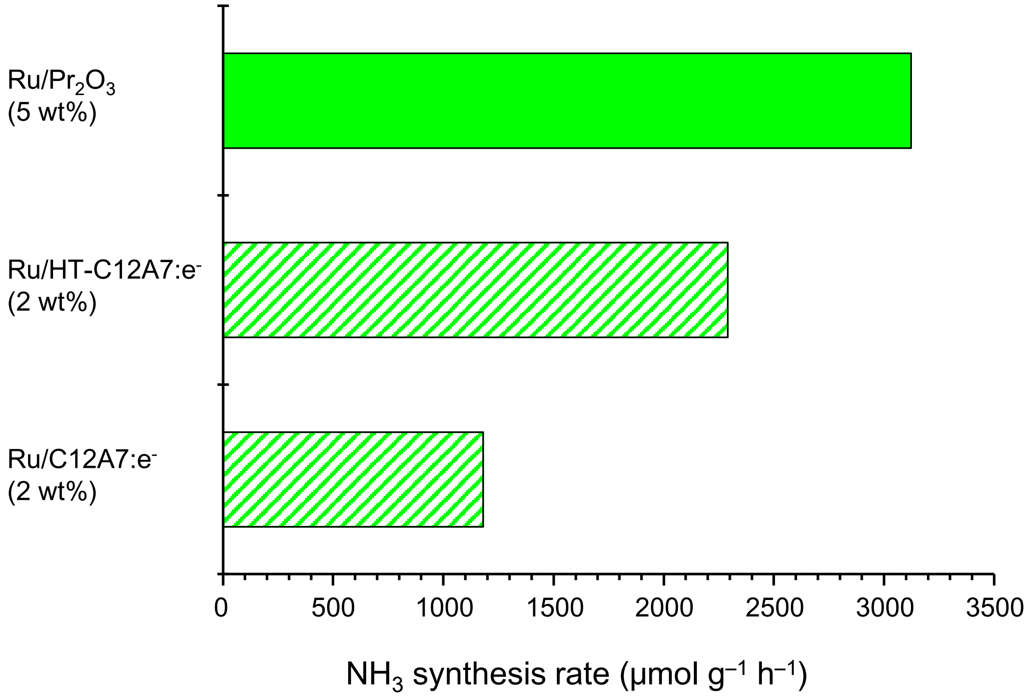

Fig. S1 Catalytic performance of supported Ru catalysts at low temperature. Reaction conditions: catalyst, 200 mg; reactant gas, $H_2/N_2 = 3$ with a flow rate of 60 mL min^{-1}; pressure, 0.1 MPa; reaction temperature, 310 °C. Ammonia synthesis rates of electride-supported catalysts were reproduced from Ref. [S3]. Kitano *et al.* improved the method of Ru/C12A7:e^- preparation [S4] and developed a highly active Ru/HT-C12A7:e^-.[S3]

2.2. *In-situ* XRD measurement

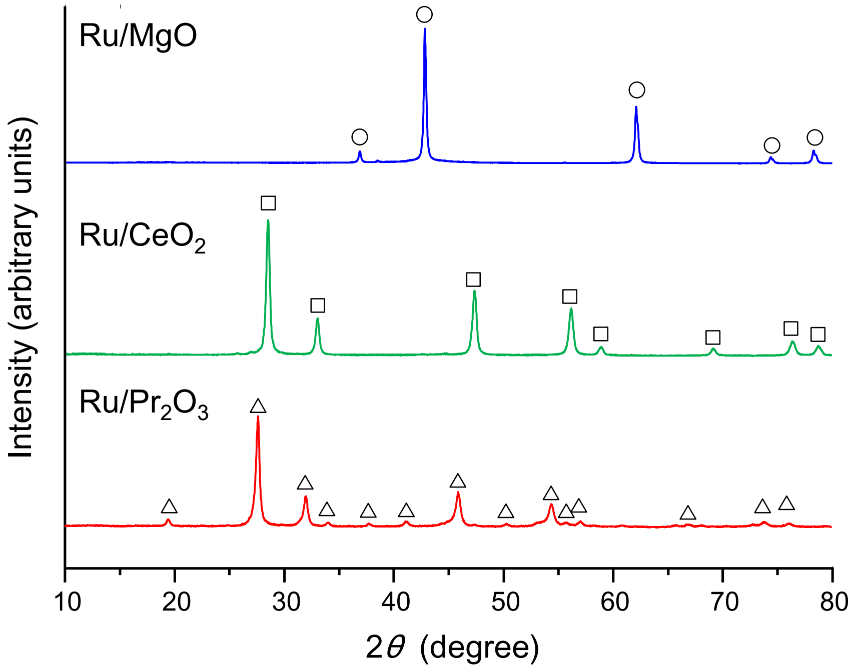

Fig. S2 *In-situ* XRD patterns of supported Ru catalysts. Samples were treated under a stream of H_2 at 400 °C and cooled to room temperature under a stream of N_2. Diffraction patterns were obtained at room temperature. ○ MgO (CSD:9013270(COD)); □ CeO_2 (CSD:028709(ICDD)); △ C-type Pr_2O_3 (CSD:NIMS_MatNavi_4295510869_1_2).

2.3. Time course of NH_3 formation over Ru/Pr_2O_3

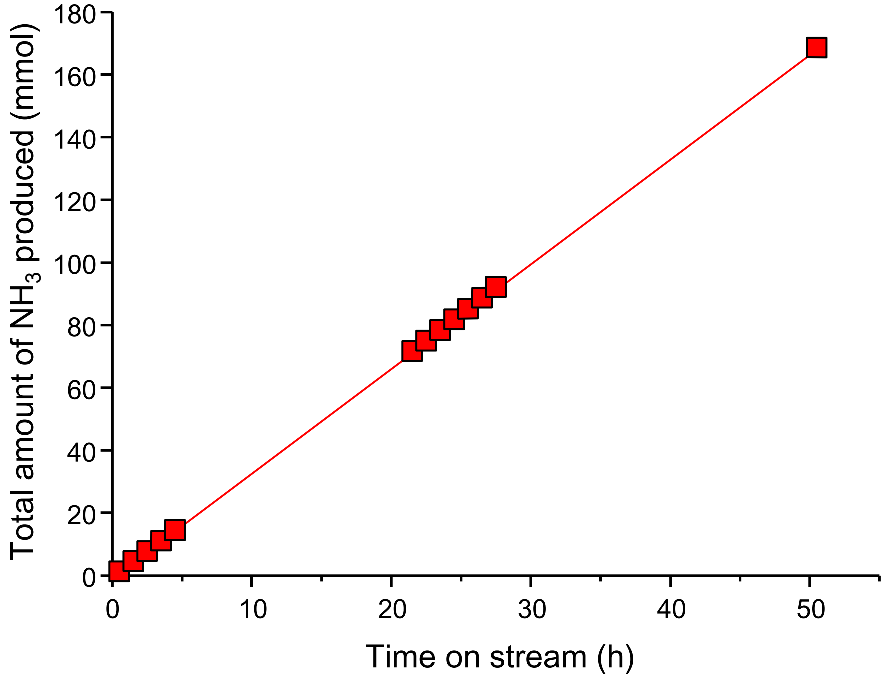

Fig. S3 Time course of ammonia formation over 5 wt% Ru/Pr_2O_3. Reaction conditions: catalyst, 200 mg; synthesis gas, $H_2/N_2 = 3$ with a flow rate of 60 mL min^{-1}; pressure, 0.9 MPa; reaction temperature, 390 °C.

2.4. STEM-EDX observation

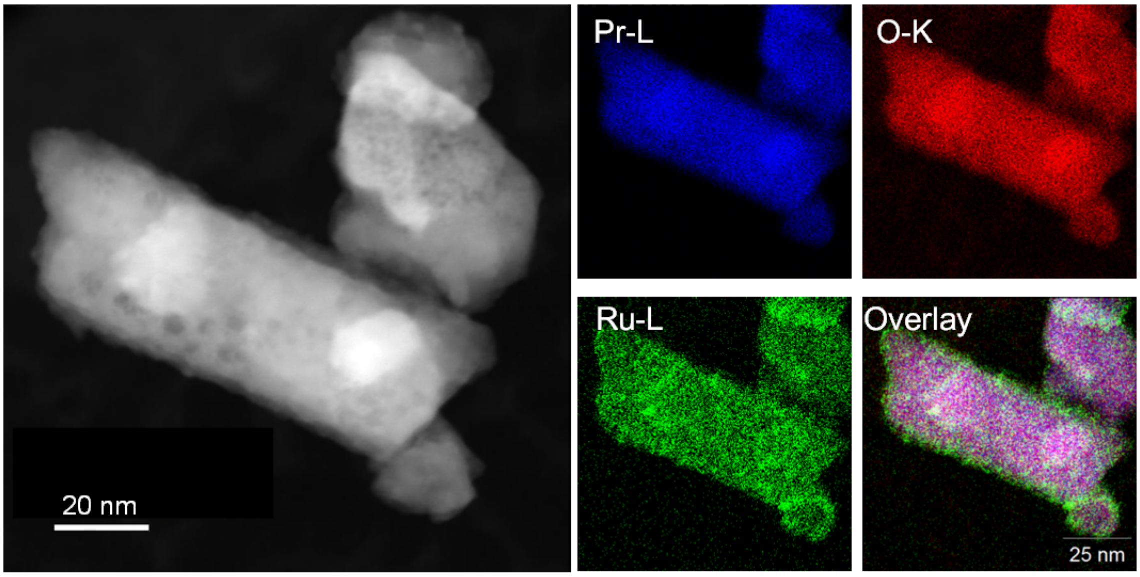

Fig. S4 HAADF-STEM image, Pr-L, Ru-K, and O-K STEM-EDX maps, and reconstructed overlay image of Pr, Ru, and O of Ru/Pr_2O_3 after H_2 reduction.

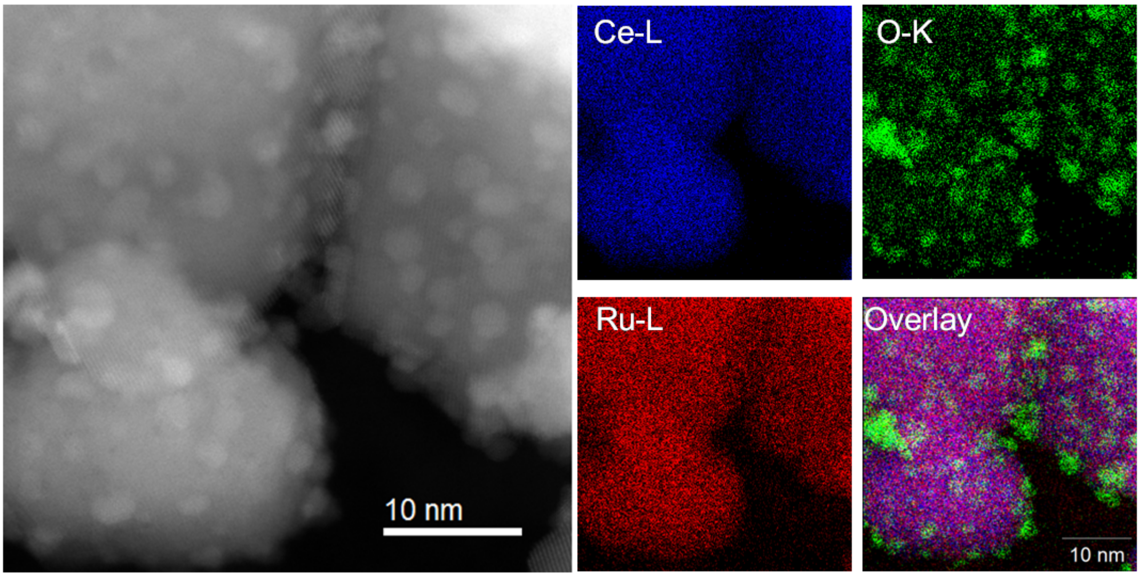

Fig. S5 HAADF-STEM image, Ce-L, Ru-K, and O-K STEM-EDX maps, and reconstructed overlay image of Ce, Ru, and O of Ru/CeO_2 after H_2 reduction.

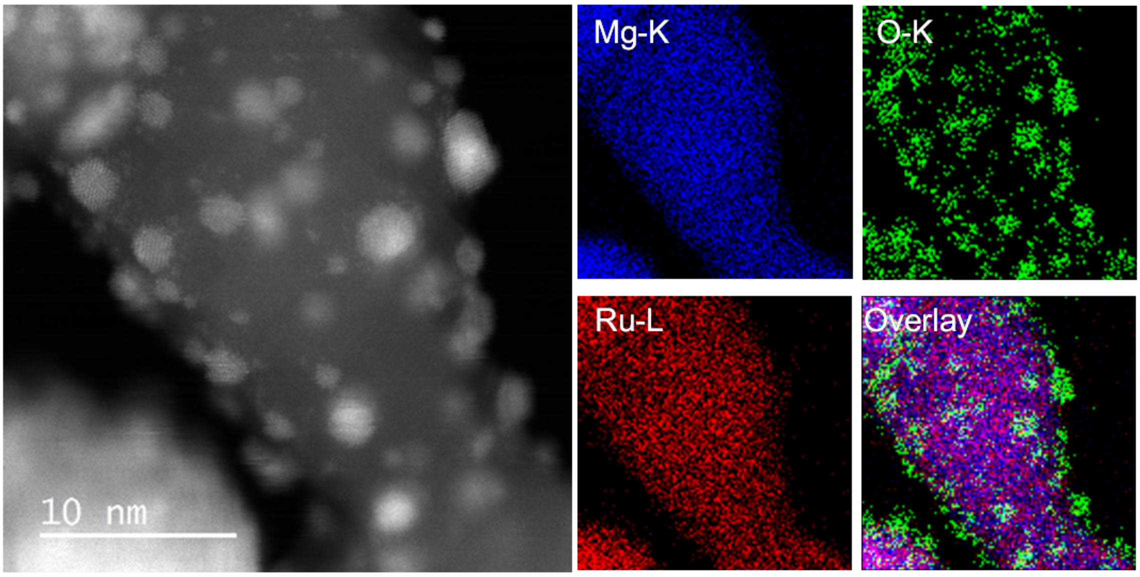

Fig. S6 HAADF-STEM image, Mg-K, Ru-L, and O-K STEM-EDX maps, and reconstructed overlay image of Mg, Ru, and O of Ru/MgO after H_2 reduction.

2.5. High-resolution TEM observations

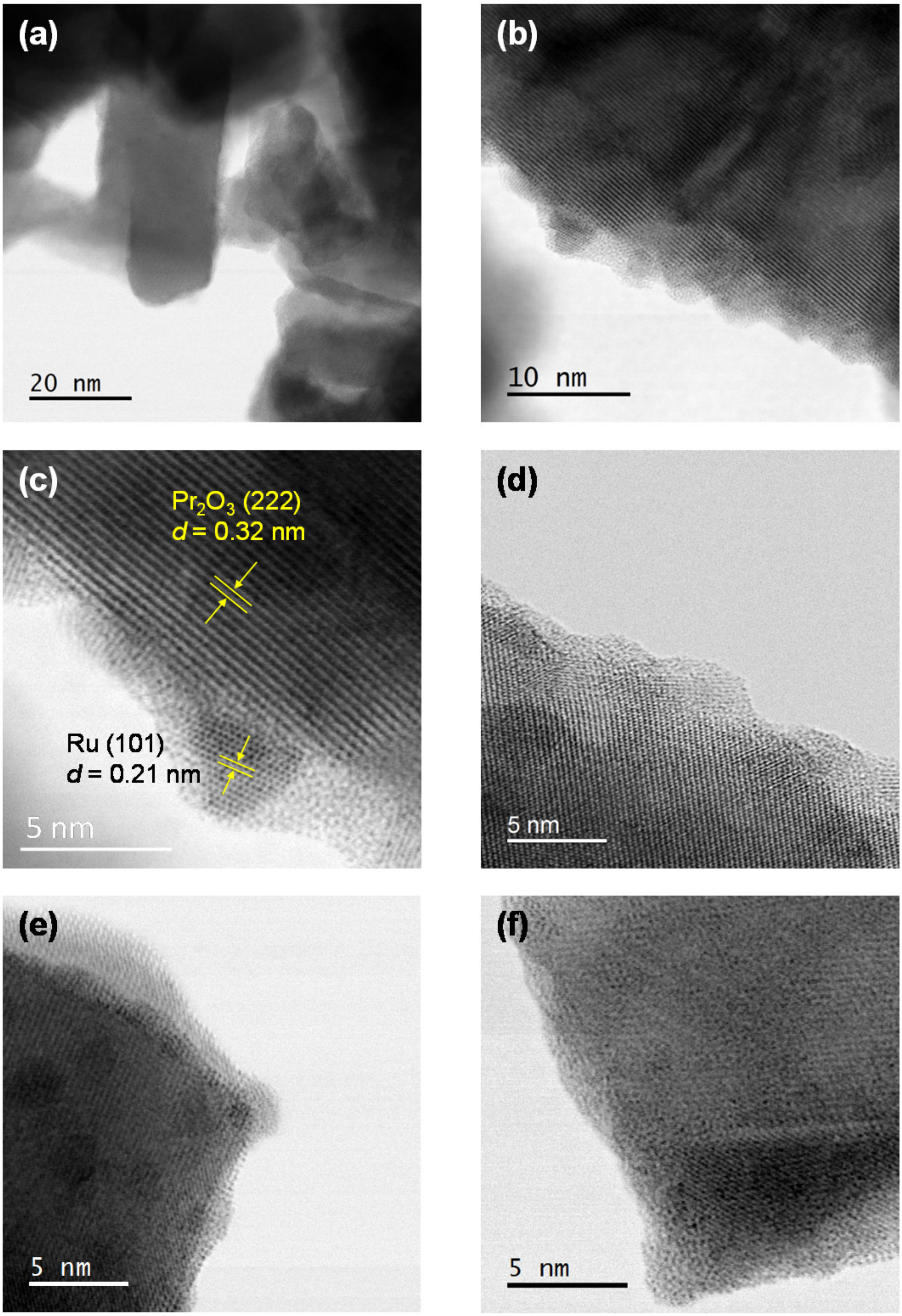

Fig. S7 High-resolution STEM images of 5 wt% Ru/Pr_2O_3.

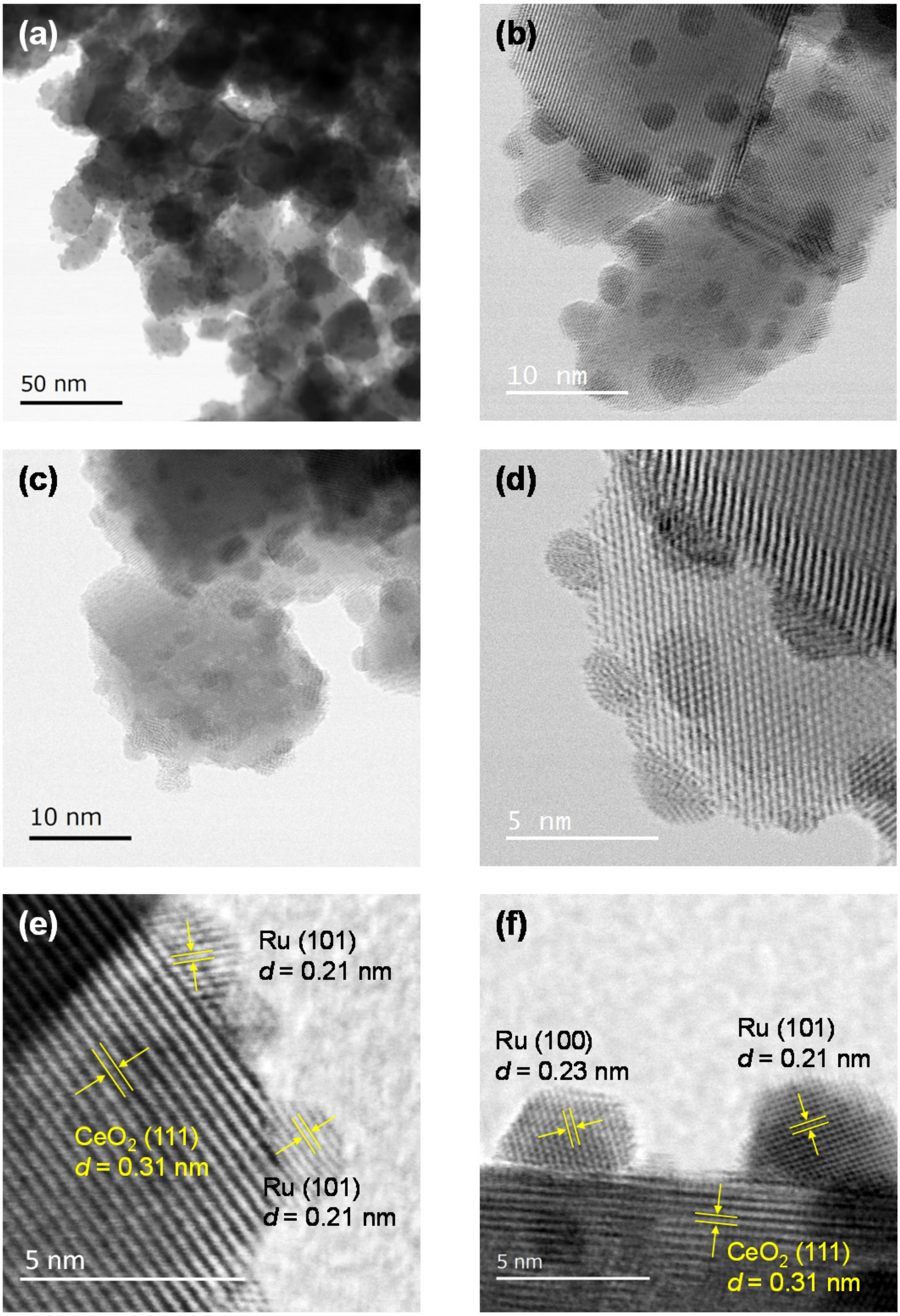

Fig. S8 High-resolution STEM images of 5 wt% Ru/CeO_2.

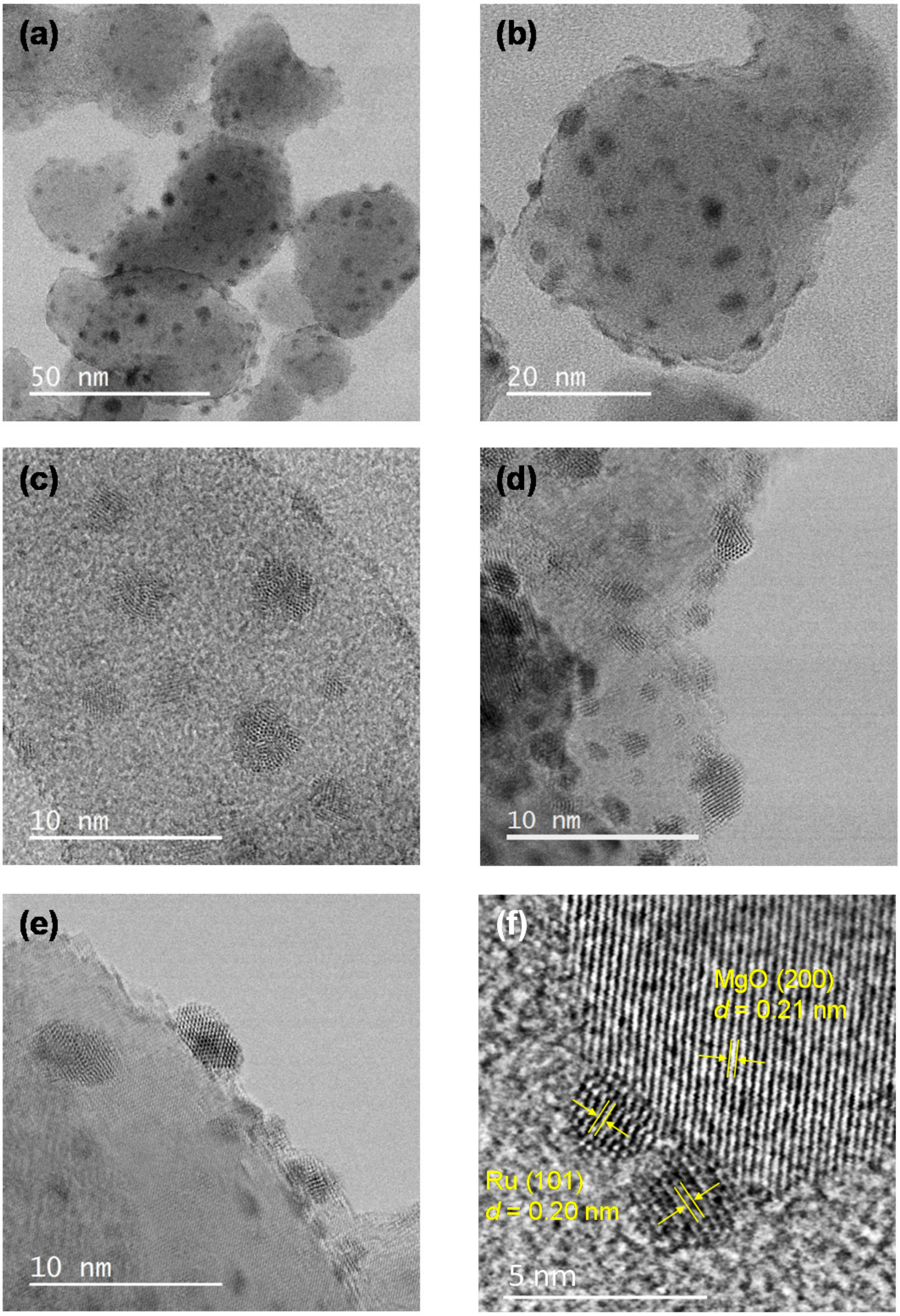

Fig. S9 High-resolution STEM images of 5 wt% Ru/MgO.

2.6. Characterization of Ru/Pr_2O_3 in different preparation stages.

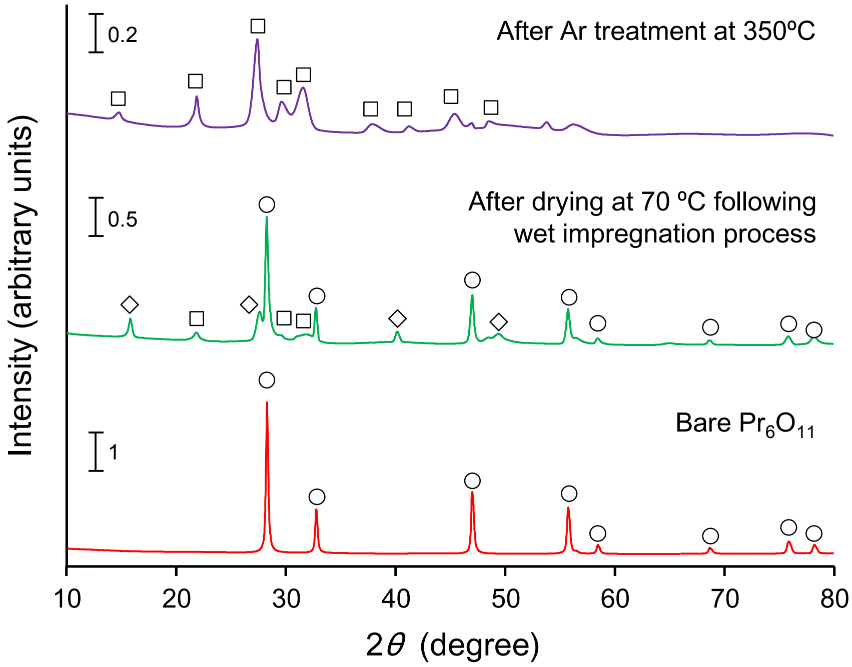

Fig. S10 XRD patterns of Ru/Pr_2O_3 in different preparation stages and bare Pr_6O_{11}. ○ Pr_6O_{11} (CSD:00-042-1121(ICDD)); ◇ $Pr(OH)_3$ (CSD:200487(ICDD)); □ PrOOH (CSD:00-027-0478(ICDD)).

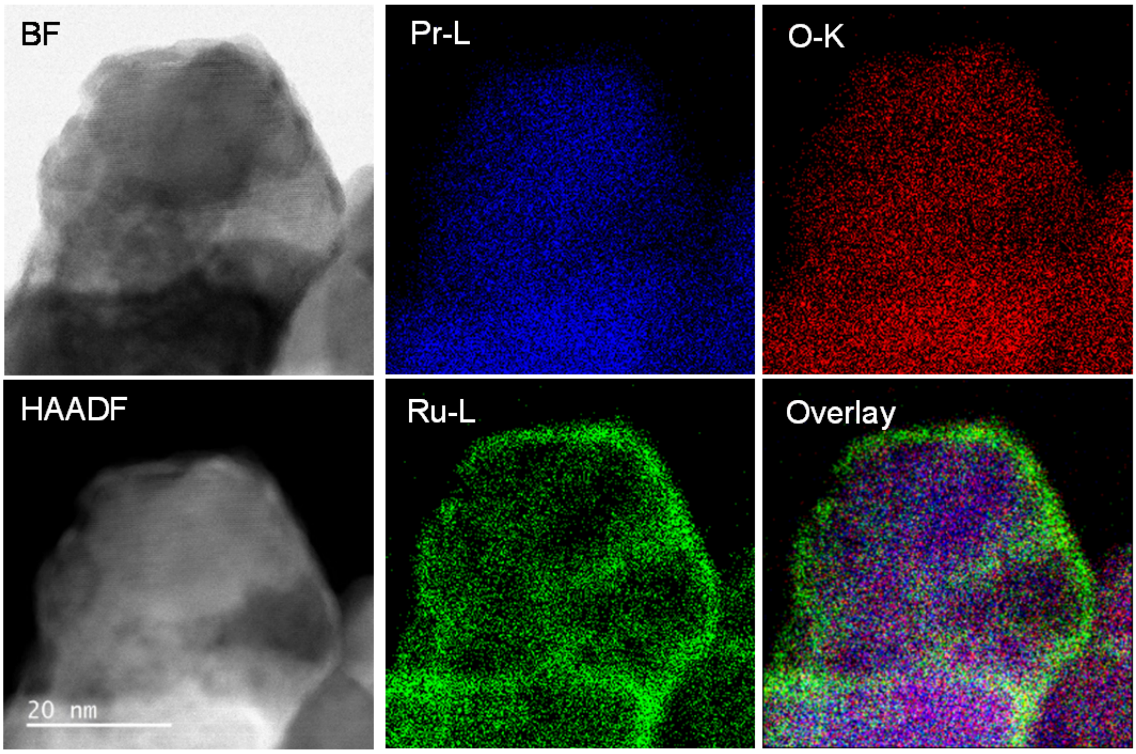

Fig. S11 BF, HAADF-STEM image, Pr-L, Ru-K, and O-K STEM-EDX maps, and reconstructed overlay image of Pr, Ru, and O of 5 wt% Ru/Pr_2O_3 after Ar treatment at 350 °C.

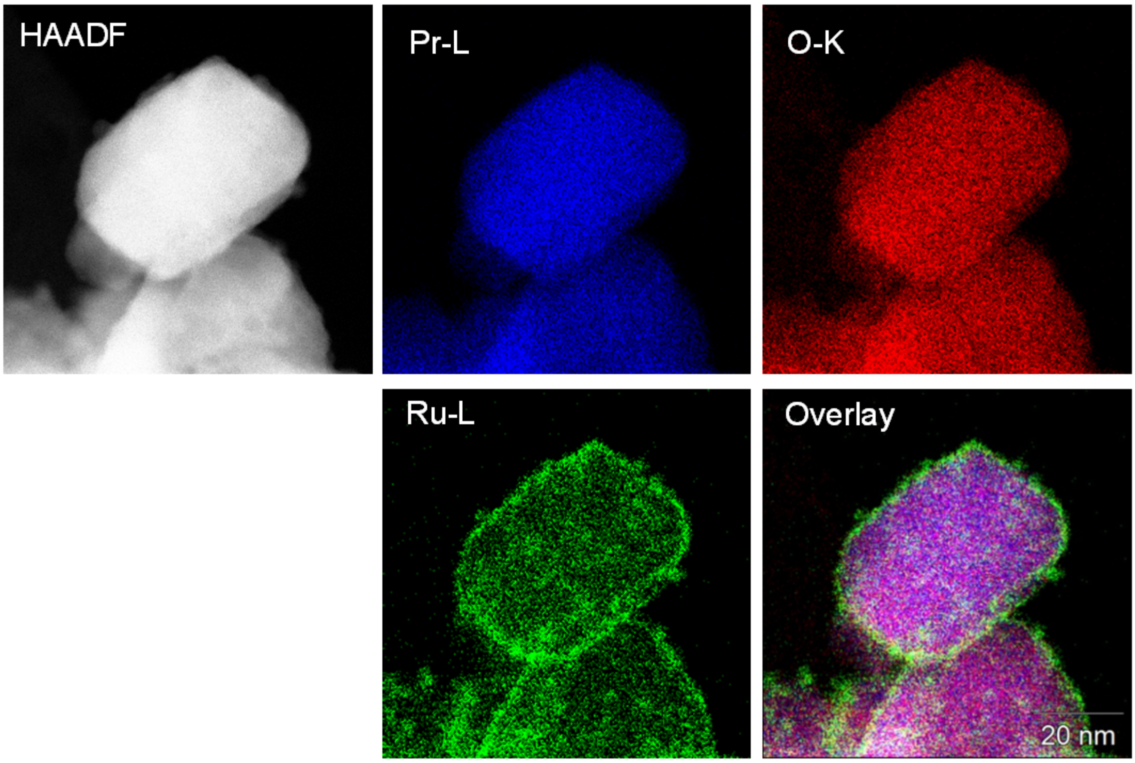

Fig. S12 HAADF-STEM image, Pr-L, Ru-K, and O-K STEM-EDX maps, and reconstructed overlay image of Pr, Ru, and O of 5 wt% Ru/Pr_2O_3 after a long-term durability test (results of activity test are shown in Fig. S3).

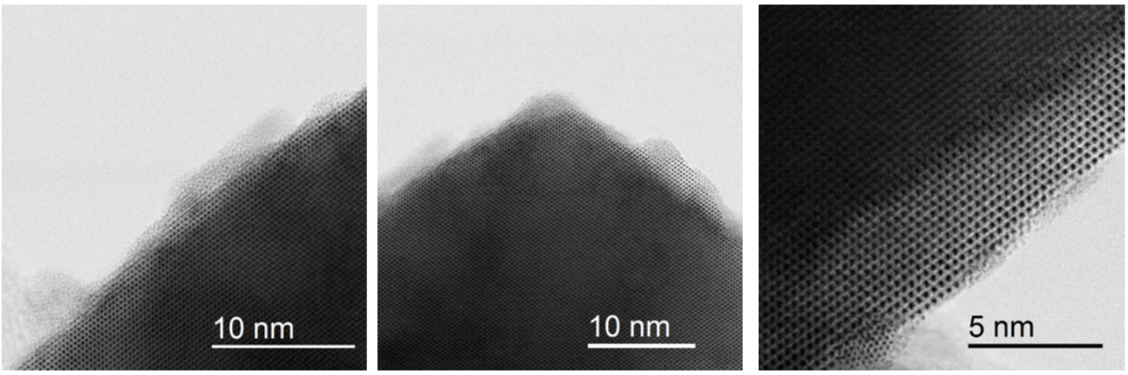

Fig. S13 High-resolution STEM images of 5 wt% Ru/Pr_2O_3 after a long-term durability test (results of activity test are shown in Fig. S3).

2.7. Characterization for basicity of the support.

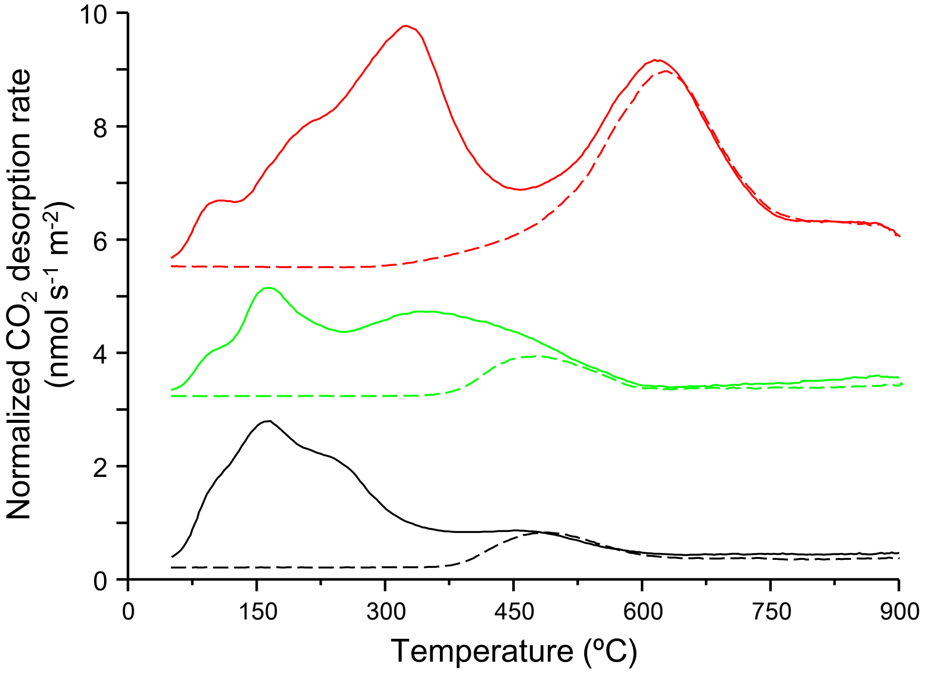

Fig. S14 CO_2-TPD profiles of supported Ru catalysts. Lines indicate desorption profiles before (- - - -) and after (——) CO_2 absorption after H_2 reduction. Intensities were normalized to the specific surface area of the catalysts. Figure 5 shows the difference between profiles.

3. References

[S1] S. Grazulis, D. Chateigner, R. T. Downs, A. F. Yokochi, M. Quiros, L. Lutterotti, E. Manakova, J. Butkus, P. Moeck and A. Le Bail, *J. Appl. Crystallogr.*, 2009, **42**, 726-729.

[S2] Y. Xu, M. Yamazaki, and P. Villars, *Jpn. J. Appl. Phys.* 2011, **50**, 11RH02.

[S3] Y. Inoue, M. Kitano, S.-W. Kim, T. Yokoyama, M. Hara, and H. Hosono, *ACS Catal.* 2014, **4**, 674-680.

[S4] M. Kitano, Y. Inoue, Y. Yamazaki, F. Hayashi, S. Kanbara, S. Matsuishi, T. Yokoyama, S. W. Kim, M. Hara, H. Hosono, *Nat. Chem.* 2012, **4**, 934-940.
